# The *h*’-Index, Effectively Improving the *h-*Index Based on the Citation Distribution

**DOI:** 10.1371/journal.pone.0059912

**Published:** 2013-04-02

**Authors:** Chun-Ting Zhang

**Affiliations:** Department of Physics, Tianjin University, Tianjin, China; Max Planck Society, Germany

## Abstract

**Background:**

Although being a simple and effective index that has been widely used to evaluate academic output of scientists, the *h-*index suffers from drawbacks. One critical disadvantage is that only *h-*squared citations can be inferred from the *h-*index, which completely ignores excess and *h-*tail citations, leading to unfair and inaccurate evaluations in many cases.

**Methodology /Principal Findings:**

To solve this problem, I propose the *h’*-index, in which *h-*squared, excess and *h-*tail citations are all considered. Based on the citation data of the 100 most prolific economists, comparing to *h*-index, the *h’*-index shows better correlation with indices of total-citation number and citations per publication, which, although relatively reliable and widely used, do not carry the information of the citation distribution. In contrast, the *h’-*index possesses the ability to discriminate the shapes of citation distributions, thus leading to more accurate evaluation.

**Conclusions /Significance:**

The *h’-*index improves the *h-*index, as well as indices of total-citation number and citations per publication, by possessing the ability to discriminate shapes of citation distribution, thus making the *h’-*index a better single-number index for evaluating scientific output in a way that is fairer and more reasonable.

## Introduction

The *h-*index, proposed by Hirsch [1], has received wide attention in recent years. For example, as of January 1, 2013, the original paper [1] putting forward the *h-*index has been cited for 1,232 times in the database of Science Citation Index (SCI). Due to its importance, the *h*-index study becomes one of the hottest topics in past years [2]. For recent reviews, refer to [3], [4], [5], [6], [7]. Although being a simple and effective index, the *h-*index suffers from drawbacks. To improve or complement the *h*-index, many *h*-type indices were proposed. We list a few of them, but not all, as follows: the g-index [8], A-index [9], R-index and AR-index [10], [11], h^(2)^- index [12], *e*-index [13], [14], etc. For more details about the *h*-type indices, refer to [3], [4], [6]. The relation among the *h*-index and some *h*-type indices was studied in [15]. One of the disadvantages of the *h*-index is the so-called isohindex problem, a phenomenon that many scientists share an identical *h*-index. To overcome the disadvantage, the real-value *h*-index [16] and rational (successive) *h*-index [17] were proposed. The time dependence of the *h*-index and *h*-type indices and the relation among them were studied in [18], [19], [20], [21]. At the same time, the *h*-index was used in various areas. For example, the *h*-index or the *h*-type indices were used for evaluating physicists [22], studying journals [23], evaluating chemical research groups correlated with peer judgment [24] and for evaluating the 100 most prolific economists [25]. Interestingly, it is reported recently that references [6], [8], [11], [22], [23] and [24] are the most-cited articles in their respective journals [2]. For example, reference [11] is the most-cited article ever, published in the *Chinese Science Bulletin* [2].

**Figure 1:**
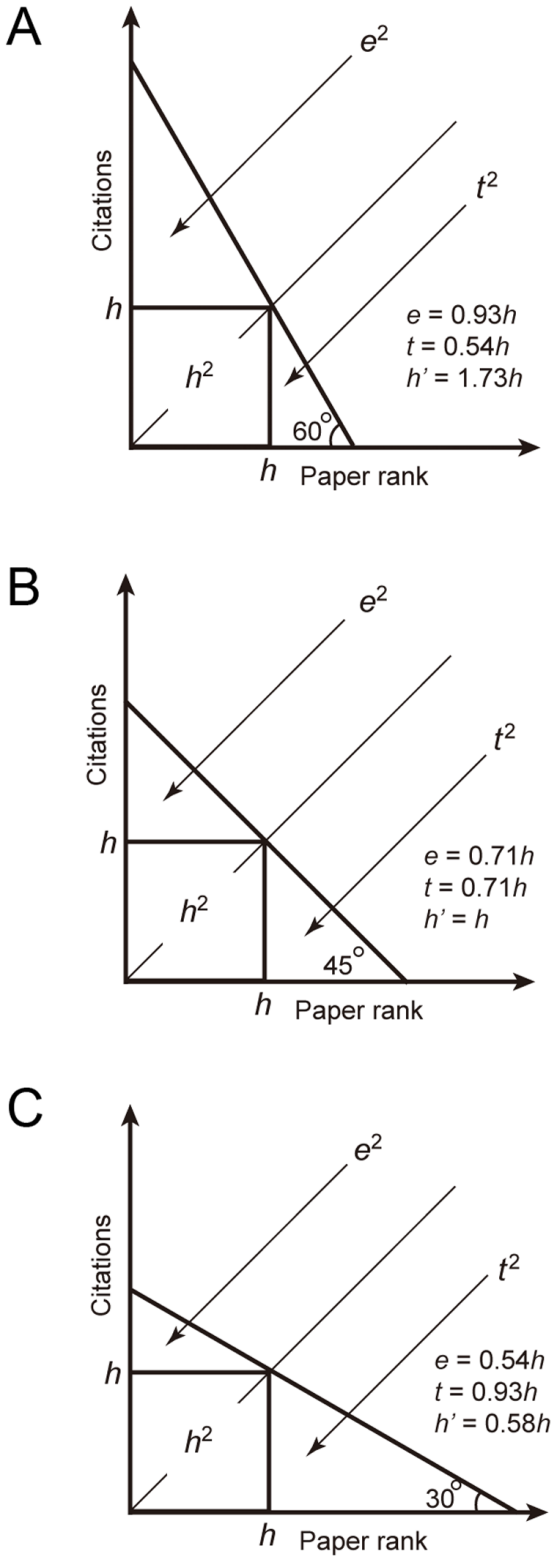
A straight-line model of the citation distribution function. The area under the citation distribution curve is divided by the *h-*index into three parts: the *h^2^*, excess citations (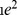) and *h-*tail citations (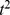). Here the citation distribution curve is simplified as a straight line. Cases shown in A, B and C all belong to an isohindex group, and they represent three types of scientists: A, perfectionist-type; B, prolific-type and C, mass producer-type [29], [30], respectively.

One important advantage of the *h-*index is its simplicity. The *h-*index uses only an integer to measure the academic output of a scientist. Therefore, the Web of Science provides the *h*-index for every scientist whose papers are indexed by the SCI database. As any single-number indicator, one of the disadvantages of the *h*-index is the loss of citation information. The area under the citation distribution is divided by the *h-*index into three parts, 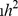, excess and *h-*tail citations (Fig. 1). The *h-*index by itself does not carry information for excess and *h-*tail citations, which can play an even more dominant role than the *h-*index in determining the shape of citation distribution curve. Ignoring the contributions from the excess and *h*-tail citations usually either under-estimates or over-estimates the academic output of the scientist under study.

The current study aims to solve the above problem by proposing the *h’-*index, a new *h*-type index that satisfies the following requirements. First, we hope to keep the most important advantage of the *h-*index, i.e., to use a single-number to measure the academic output of scientists. Second, the new index should carry the main information of citation distributions. In other words, in addition to 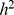, the new index should reflect the information from the excess citations [13] and *h-*tail citations [26], [27]. Third, as a single-number evaluation index, the total citation number and citations per publication are widely used to evaluate the academic performance of scientists nowadays, e.g., the latter was used to rank the world’s top 100 materials scientists by Thomson- Reuters in 2011 [28]. Therefore, we hope that the new index should be highly and linearly correlated with these two indices, respectively, while overcoming their main disadvantage of having no information about citation distribution.

## Results and Discussion

### Definition of the 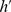-index

As pointed out previously, the area under the citation distribution function is divided by the *h-*index into three parts, representing the *h^2^*, excess and *h-*tail citations. However, the shapes of the citation distributions are different for different scientists. The shapes may be roughly divided into three types, represented by a simple straight line model (Fig. 1).

The distribution functions shown in Fig. 1A, B and C represent three types of scientists.

Scientists are roughly divided into 3 types [29], [30]. Scientist A is called a perfectionist, who has few publications, which, however, are highly cited. Scientist B is called a prolific-type scientist, who publishes a large number of papers which also tend to be highly cited. Scientist C is called a mass producer, who publishes a large number of papers that are lowly cited. Scientist A corresponds to Fig. 1A, where 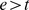 or 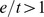, with 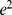 and 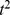 corresponding to excess and *h-*tail citations [13], [26], [27]. Here the *e*-index and *t*-index need to be explained. The *e*-index is the square root of the excess citations over 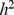 in the *h*-core [13]. The *t*-index is the square root of the *h*-tail citations [26], [27]. Scientist B corresponds to Fig. 1B, where 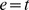 or 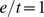 and scientist C corresponds to Fig. 1C, where 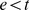 or 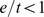.

Based on the above analysis, it can be seen that the real number 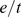 is an important parameter to characterize the shapes of the citation distributions. Letting
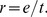
(1)


We call *r* the *e*–*t* ratio, or the head-tail ratio. The three cases of 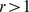, 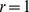 and 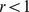 correspond to three types of the citation distribution functions. The *e*–*t* ratio, 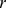, is an important index to capture the overall shapes of citation distribution functions. The shapes of citation distribution functions for 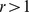 are peaked, and for 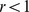 the shapes of the citation functions are flat with a long tail, whereas for 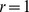 the citation functions are roughly symmetrical with respect to the diagonal line of the coordinate system. When 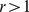, especially 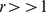, the *h-*index under-estimates the academic output of the scientist being studied, whereas when 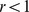, especially 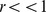, the *h-*index over-estimates the academic output of the scientist being studied. When 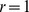, the *h-*index properly reflects the academic output of the scientist under study. To have a fair evaluation of the academic output of scientists, we propose a novel *h*-type index, the 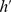-index, which is defined by
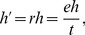
(2)where *e*, *h* and *t* are the *e*-index, *h-*index and *t-*index, respectively.

The citations received by all papers in the *h-*core, denoted by 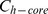, are
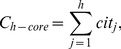
(3)where 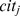 are the citations received by the *j* ^th^ paper. Letting 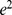 denote the excess citations within the *h-*core, we find [13]

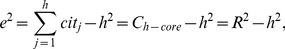
(4)where R is the R-index [11]. Thus,



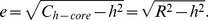
(5)Meanwhile, the *t-*index was defined by [27]

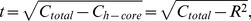
(6)where 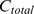 is the number of total citations received by all papers published by the scientist under study. Finally, we have 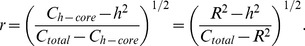
(7)


### Comparisons between the h’-index and h-index

First, two concrete examples are considered. According to the citation information provided by Dodson [14], 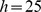, 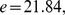 and 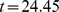, we find 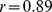 and 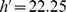. Therefore, the *h-*index for Dodson is properly applicable. Another example is for the chemist Berni Alder, where 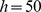, 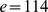 and 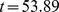 [13], we find 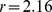 and 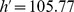. Therefore, this example shows that Alder’s *h-*index severely under-estimates his academic output, whereas the 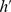-index gives him a relatively fair evaluation.

Second, we turn to study the research output of three types of scientists A, B and C within the same isohindex group. The three scientists are the real applicants applying for the Young Investigator Programme, in the program of the European Molecular Biology Organization (EMBO) in Heidelberg, Germany [29]. Scientist A, B and C belong to the perfectionist-type, prolific-type and mass producer-type scientist, respectively [29], [30].

The common character of them is that they all have the same *h*-index, 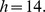 Based on the data provided by Fig. 1 in [29], the number of papers, the total number of citations, the citations per publication, the *e*-index, *t*-index, the *e*-*t* ratio *r* and the *h*’-index are all listed in Table 1. As we can see that the academic performance of the three types of scientists A, B and C is quite different. This example shows that the *h*-index does not posses the ability to discriminate different shapes of the citation distributions. In contrast, the citations per publication and the *h*’-index appropriately reflect the academic performance of the three types of scientists A, B and C, whereas the *h*-index does not discriminate the three types of scientists correctly.

**Table 1:** Comparisons among three types of scientists, A, B and C in the same isohindex group with h = 14^a^.

Type	Cit	P	Cit/P	e	t	r	h’-index	h-index
A	1321	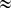 20	66.05	32.91	6.30	5.23	73.19	14
B	408	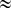 20	20.40	12.61	7.28	1.73	24.25	14
C	592	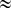 87	6.80	7.69	18.37	0.42	5.86	14

a The symbols Cit, P and Cit/P denote the total number of citations, the number pf papers and the citations per publication. The indices *e*, *t*, *r* and *h*’-index are defined in eqs. (5), (6), (1) and (2), respectively. The values of Cit were provided by Fig. 1 of [29]. Note that the figures of P were appropriately estimated from Fig. 1 in [29]. The indices *e* and *t* were calculated from the values of 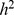 upper and 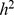 lower, respectively, provided by Fig. 1 of [29], via the formulas 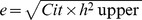 and 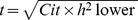.

**Figure 2:**
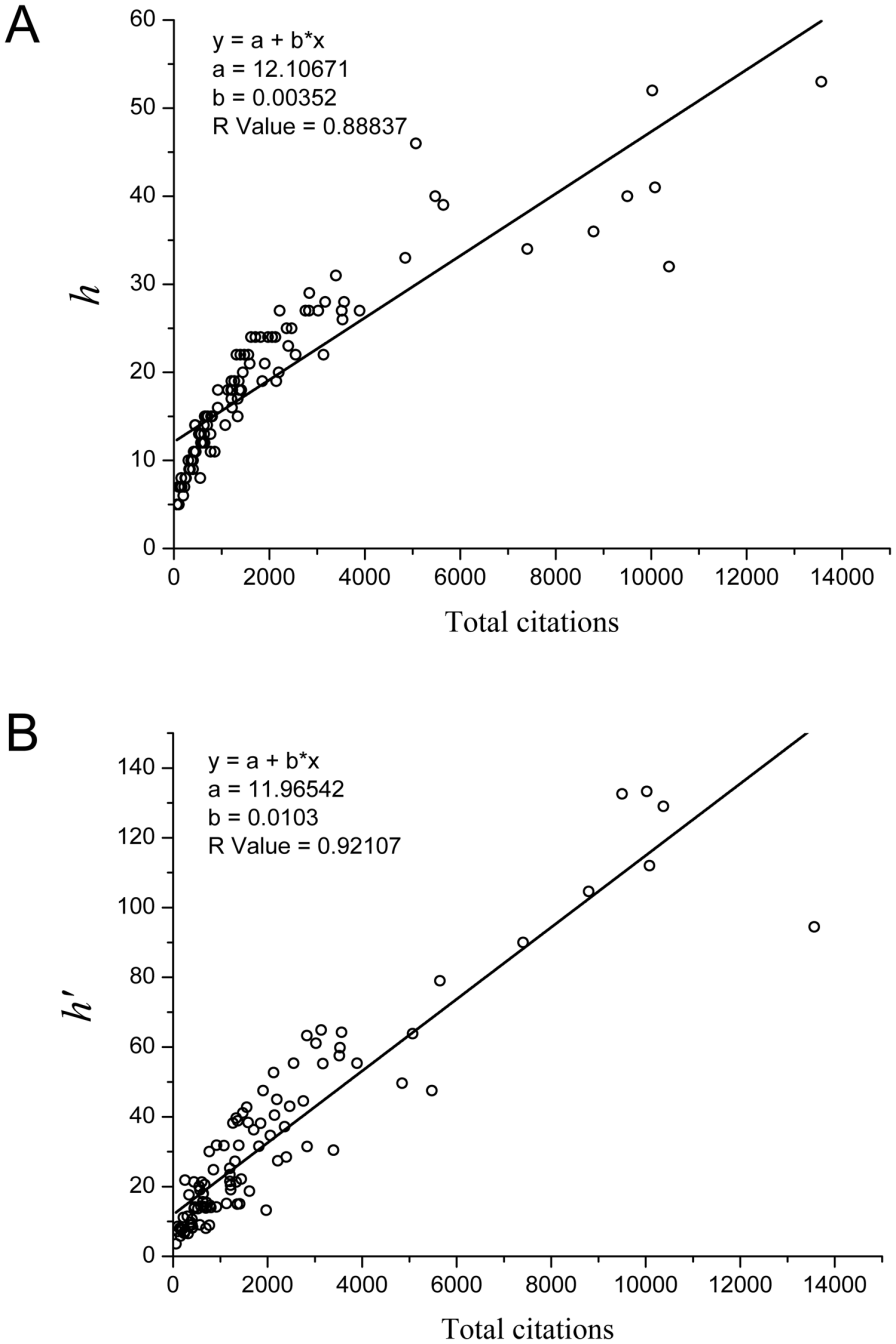
The correlation between the *h-*index and the 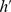-index with the number of total citations. A, the correlation between the *h-*index and the number of total citations, and B, the correlation between the 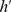-index and the number of total citations, based on the data of the 100 most prolific economists. Note that the 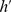-index shows better linear correlation with the number of total citations than the *h-*index.

**Figure 3:**
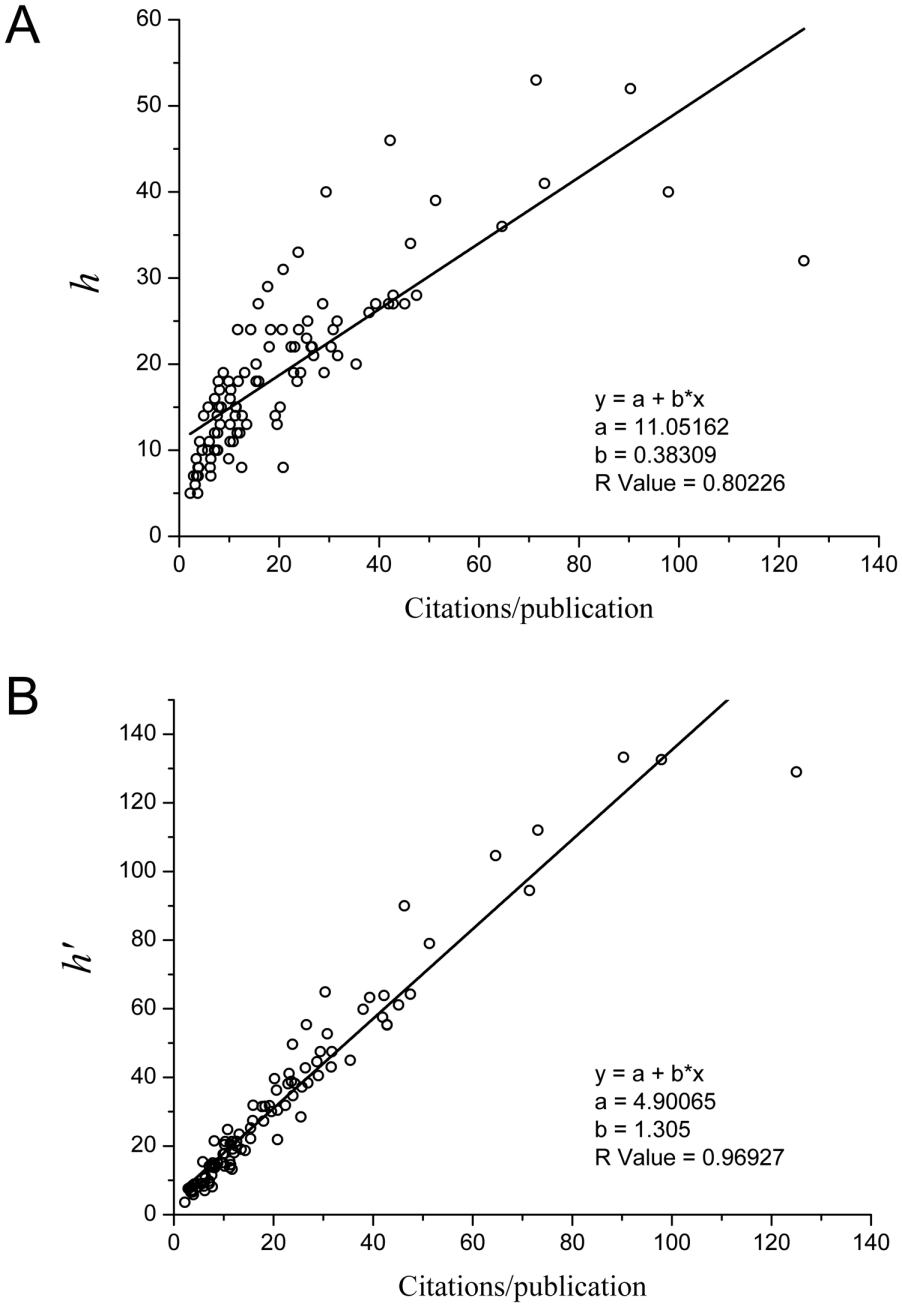
The correlation between the *h-*index and the 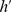-index with the index of the citations per publication. A, the correlation between the *h-*index and the index of the citations per publication, and B, the correlation between the 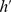-index and the number of total citations, based on the data of the 100 most prolific economists. Note that the 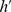-index shows better linear correlation with the index of citations per publication than the *h-*index.

Third, in what follows, we compare the *h-*index and 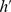-index for the 100 most prolific economists [25]. The correlation between the *h-*index and total-citation number for the 100 economists is shown in Fig. 2A, whereas that between the 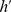-index and total- citation number is shown in Fig. 2B. It is seen that the 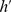-index has a linear correlation with the total-citation number better than the *h-*index. Likewise, the linear correlation between the *h-* or *h’-*index and citations per publication for the 100 economists is shown in Fig. 3A and B, respectively. We can see that the 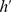-index has a higher linear correlation with the citations per publication than the *h-*index. Of note, the correlation coefficient between the 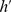-index and the citations per publication is as high as 0.969. However, this does not imply that the two are nearly equal with each other. In fact, the average value and standard deviation of the 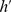-index and citations per publication over the 100 most prolific economists are 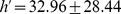 and 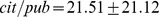, respectively.

Theoretically, the 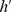-index carries more citation information than the *h-*index, because the *h-*index captures only the information of the *h^2^* citations, whereas the 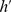-index captures not only the *h^2^* citations, but also the excess and *h-*tail citation information. Examples shown in Fig. 1 and Table 1 indicate that the *h-*index does not possess the ability to discriminate the shapes of the citation distribution functions. The three cases shown in Fig. 1 belong to the same isohindex group, i.e., they have an identical *h-*index. The 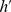-index properly discriminates the three cases, with 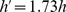, 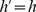 and 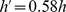, respectively, for the cases shown in Fig. 1A, B and C.

As mentioned above, the *h*-index has the isohindex problem. Since the *h*’-index is a real number, it provides an alternative solution in addition to those suggested in [16] and [17]. However, in some special cases, the *h*’-index can have the similar problem. For instance, if the citation curve in Fig. 1A is modified so that it is not linear, with the area of upper part decreasing to 0.54 *h*, while the lower part (*h*-tail) remains the same 0.54 *h,* then *e = *0.54 *h* = *t* and *h*’ = *h*, as in Fig. 1B. This example shows that different citation distribution may lead to the same *h*’-index. However, it seems that this problem of the *h’*-index occurs so rarely that it is highly unlikely in reality. In addition, it is also possible that different *h*-values can lead to the same *h’*-values, but it is not yet observed.

Hirsch suggested that the *h-*index is preferable to single-number criteria commonly used to evaluate scientific output of a researcher, such as the number of total citations and citations per publication [1]. Here we show that as a single-number evaluation index, the 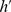-index is also superior to the number of total citations and citations per publication. First, we compare the *h*’-index with the number of total citations. As a single-number, the merit of the number of total citations is to measure the total output of the scientist under study using only a simple integer. However, its notable disadvantage is that this number cannot discriminate the shapes of the citation distributions. Refer to Fig. 1 A and C, where the numbers of total citations are identical, being equal to the total area under the citation distribution functions. However, our scientific common sense shows that scientist shown in Fig. 1A (perfectionist with few but influential papers) has a better academic performance than that in Fig. 1C (mass producer with many low-impact papers). This fact is correctly reflected by the 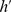-index, but not by the number of total citations. Second, in terms of citations per publication, although this widely used index seems better than the number of total citations, it also does not possess the ability to discriminate the shapes of the citation distribution functions. Consequently, as correctly pointed out by Hirsch [1], this index (cit/pub) usually rewards low productivity, and penalizes high productivity.

### Ranking of the Performance of Scientists Based on Various Evaluation Indices

Here we show as an example rankings of the academic performance of the 100 most prolific economists. For convenience, we list only the top ten of the rankings (Table 2). The rankings are based on the 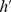-index, citations per publication and the *h-*index, respectively. As we can see from Table 2 that the top three economists based on the 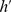-index were A Shleifer, RJ Barro and RF Engle, whereas the top three based on the citations per publication were R F Engle, R J Barro and A Shleifer. That is to say, except the ranking order is slightly different, the names of the top three were identical. However, the top three economists based on the *h-*index were different from those based on the 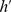-index and cit/pub. Indeed, except A Shleifer, the names of the remaining two were different.

**Table 2:** The top ten prolific economists based on different evaluation indices.

No.	Ranking based on *h’*	*h’*	Ranking based on *cit/pub*	*cit/pub*	Ranking based on *h*	*h*
1	A. Shleifer	133.3	R.F. Engle	125.0	J.E. Stiglitz	53
2	R.J. Barro	132.6	R.J. Barro	97.9	A. Shleifer	52
3	R. F. Engle	129.0	A. Shleifer	90.3	J. Tirole	46
4	J.J. Heckman	112.0	J.J. Heckman	73.1	J.J. Heckman	41
5	C.W.J. Granger	104.6	J.E. Stiglitz	71.4	M.S. Feldstein	40
6	J.E. Stiglitz	94.5	C.W. J. Granger	64.6	R.J. Barro	40
7	P.C.B. Phillips	90.0	L.H. Summers	51.3	L.H. Summers	39
8	L.H. Summers	79.0	O. Blanchard	47.5	C.W. J. Granger	36
9	J. Tobin	64.9	P.C.B. Phillips	46.3	P.C.B. Phillips	34
10	O. Blanchard	64.3	A.B. Krueger	45.1	P.A. Samuelson	33

Among the top ten of the 100 most prolific economists based the 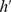-index, nine were identical with those based on the citations per publication, indicating that the two rankings were considerably consistent. In contrast, of the top ten economists ranked on the 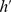-index, only seven were identical with those based on the *h-*index. Furthermore, Dr. RF Engle, who ranked No. 1 based on cit/pub, and No. 3 based on the 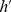-index, did not appear in the name list of the top ten economists based on the *h-*index. Of the evaluation indices currently available, it is believed that the index of citations per publication is a relatively reliable index for ranking the academic performance of scientists. Recently, the index of citations per publication was used to rank the world’s top 100 materials scientists by Thomson-Reuters [28]. In the analysis above, we show that the 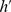-index is preferable to citations per publication, because the index of citations per publication does not possess the ability to discriminate the shapes of the citation distribution functions. As a consequence, the index of citations per publication usually rewards low productivity, and penalizes high productivity [1]. Our overall opinion is that the 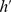-index is one of the best single-number evaluation indices, which can be used to rank the academic performance of scientists in a way that is fairer and more reasonable.

### A Study Based on a Power Law Model

Based on the citation distribution function 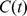, the *e*–*t* ratio can be calculated. Here we study only a simple power law model known as a Lotka’s model. We assume that
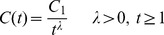
(8)where 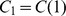 is the maximum citations received by the papers in the *h-*core. This type of function is in fact the Zipf-type formulation rather than the Lotka [31]. It was shown that [31]



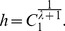
(9)Please refer to equations (8) and (9) of Reference [31]. We further assume 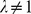. According to the definition of the *h-*index [1], we have 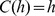, leading to the above result eq. (9) [13]. Based on eqs. (4) and (9), we find [13]

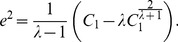
(10)


The *t-*index can be calculated by.
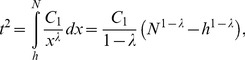
(11)where *N* is the number of all papers published by the scientist under study. Consequently, we have



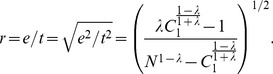
(12)It can be seen that the parameter 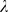 is an important factor to determine the *e*-*t* ratio 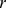. The parameter 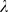 cannot be too large. For example, letting 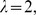 and assuming 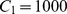,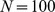, we find 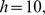 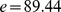 and 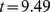. Consequently, 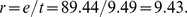 This result shows that even when 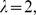 the excess citations (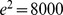) and *h-*tail citations (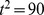) cannot be ignored. As a result, the 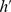-index (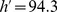) results in a more reasonable evaluation.

**Figure 4:**
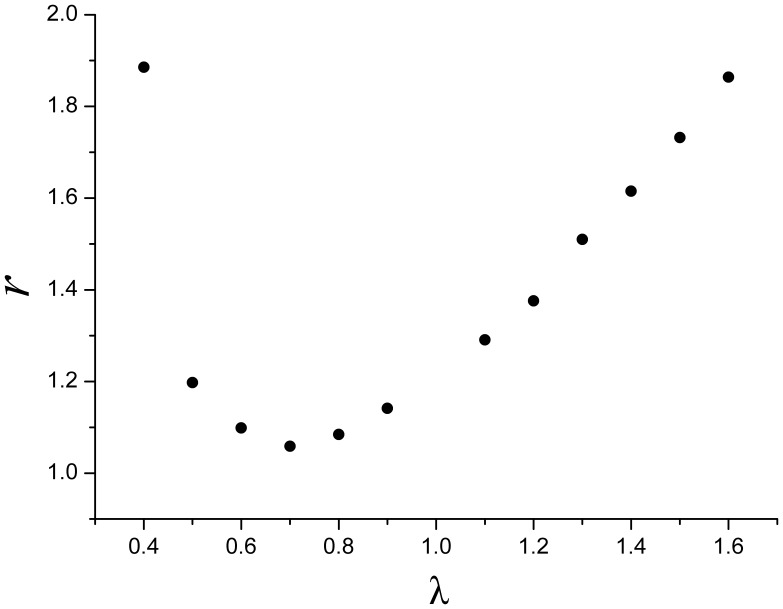
A numerical example of the *e*-*t* ratio 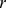. Based on a power law model, the *e*-*t* ratio 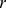 can be calculated. Letting 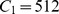,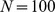 and 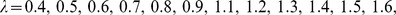 respectively, we calculate the values of the *e*-*t* ratio 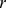 for each case. Using eq. (12), we find 12 values of 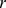, which are shown as a function of 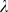. This example shows that the power parameter 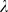 is one of the key factors to determine the *e*–*t* ratio 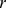.

To have an intuitive picture, let us consider some numerical examples as follows. Taking 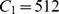, 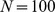 and letting 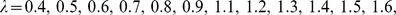 respectively, we calculate the value of 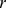 for each case. Using eq. (12), we find the coordinates of 12 points in the plane of 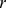 *versus* 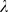, as shown in Fig. 4. This example further shows that the power parameter 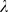 is one of the key factors to determine the value of the *e*-*t* ratio 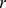.

### Concluding Remarks

The three commonly used single-number indices, the *h-*index, total-citation number and citation per publication, all suffer from critical drawbacks. (i) Because of the loss of the information of citation distribution, evaluations based on the *h-*index alone can be misleading, as exemplified by data shown in Table 1. (ii) The total-citation number does not carry the information of the citation distribution, as reflected by the typical examples shown in Fig. 1A and C. Although both cases correspond to the same number of total citations, the shapes of the citation distribution are quite different. (iii) Likewise, the index of citations per publication also does not possess the ability to discriminate the shapes of the citation distribution functions. As a consequence, the index (cit/pub) usually rewards low productivity, but penalizes high productivity [1].

The *h’-*index appears to overcome the above drawbacks by carrying additional information derived from citation distribution. In summary, the *h’-*index has the following features. (i) It is highly consistent with indices of total-citation number and citations per publication, which are relatively reliable and thus widely used in the evaluation of the academic output of scientists currently. (ii) Compared to the total-citation number and citations per publication, the *h’-*index possesses the ability to discriminate the shapes of the citation distributions, and thus leading to more reasonable evaluation. (iii) Compared with the *h-*index, the 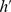-index appropriately carries the information of the excess and *h-*tail citations. (iv) The 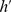-index is a real number, thus largely solving the problem of isohindex groups of the *h-*index. In conclusion, these features enable the *h’-*index to be a better single-number index for evaluating scientific output in a way that is fairer and more reasonable.

## Materials and Methods

The data used were from [25]. Please refer to Table A1, on pp.323–324 of [25]. The calculations performed in this paper were simple and trivial.
